# Natural microbial exposure populates the maternal fetal interface with diverse T cells

**DOI:** 10.3389/fimmu.2025.1616491

**Published:** 2025-07-09

**Authors:** Amy Whillock, Perianne Smith, Sarah Burger, Adhvaith Sridhar, Alex Lindgren, James Berg, Sayaka Tsuda, Shweta Mahajan, Tamara Tilburgs, Nathaniel J. Schuldt

**Affiliations:** ^1^ Department of Pediatrics, University of Minnesota Medical School, Minneapolis, MN, United States; ^2^ Center for Immunology, University of Minnesota, Minneapolis, MN, United States; ^3^ Microbiology, Immunology, and Cancer Biology, University of Minnesota, Minneapolis, MN, United States; ^4^ College of Veterinary Medicine, University of Minnesota, Saint Paul, MN, United States; ^5^ Division of Immunobiology, Cincinnati Children’s Hospital Medical Center, Cincinnati, OH, United States; ^6^ Department of Obstetrics and Gynecology, University of Toyama, Takaoka, Japan; ^7^ Departrment of Pediatrics, Cincinnati Children’s Hospital Medical Center, Cincinnati, OH, United States; ^8^ Center for Inflammation and Tolerance, Cincinnati Children’s Hospital Medical Center, Cincinnati, OH, United States; ^9^ Masonic Cancer Center, University of Minnesota, Minneapolis, MN, United States

**Keywords:** pregnancy, T cells, maternal fetal interface, dirty mice, microbial exposure, placenta, decidua

## Abstract

Diverse T cell types accumulate at the human maternal fetal interface (MFI) during pregnancy to orchestrate immune tolerance for foreign fetal/placental tissues and immunity to pathogens. Yet, the dynamics of T cell influx and function at the MFI remain poorly defined. Conventional specific pathogen free (SPF) murine models fail to replicate the number and diversity of T cells in the human MFI, hindering mechanistic study of MFI T cells. Here we present an innovative use of a natural microbial exposure (NME) mouse model that enhances T cell influx and diversity in the MFI. We defined changes in the MFI of NME mice, relative to SPF mice and human tissues using transcriptomic and proteomic approaches. Physiological maternal microbial burden reproduced key features of human MFI immunology by i) significantly increasing the numbers and diversity of CD4 and CD8 effector and memory T cells at the MFI; ii) skewing the CD8 T cell composition towards tissue resident memory phenotypes with increased signatures of activation and dysfunction similar to human decidual T cells; and iii) expanding unconventional γδ T cells and Killer Lectin-like Receptors (KLR) expressing T cell types at the MFI, representative of an enhanced ability to interact with placental trophoblasts or infected cells. Thus, maternal microbial exposure induces vast changes to T cell numbers, diversity and functions at the MFI that models human MFI T cells with great fidelity. The NME model allows for improved translational investigation of the mechanisms of T cell tolerance, immunity, and inflammation in pregnancy.

## Introduction

Balancing the requirements for host defense and tolerance to fetal antigens is critical for successful pregnancy outcomes (1, 2). A loss of placental immunity or tolerance can drive excessive placental inflammation, which is strongly associated with spontaneous preterm birth, fetal growth restriction (FGR), stillbirth, or congenital infection (3–5). T cells found at the maternal-fetal interface (MFI) play a central role in executing both antimicrobial and immune regulatory functions. Regulatory T (T_REG_) cells accumulate in decidual tissues of pregnant mice and humans; TREG cells have been shown to restrain immune responses to fetal allo-antigens in mice (6–8) and humans (9, 10). In addition, T_REG_ frequencies and functions were shown to be diminished during placental inflammation and complications of pregnancy (11–14). By contrast, cytotoxic CD8 T cells are effective killers of cells expressing foreign antigens, including microbial or fetal allo-antigens (2, 15, 16). Paradoxically, despite the inherent risk of cytotoxic T cell responses, memory CD8 T cells accumulate at the MFI over the course of gestation in humans, becoming the most abundant immune cell at the MFI late in the third trimester (17–23). In addition, both decidual CD8 T cells with fetal and viral specificity expressing markers of T cell activation and dysfunction were found (22). In contrast to humans, CD8 T cells are rare in uterine tissues of conventional specific pathogen free (SPF) mice during healthy pregnancy, possibly due to epigenetic silencing of key T cell attracting chemokines in these mice (24). The paucity of CD8 T cells at the MFI in SPF mice limits their utility for immunologic study, underscoring the critical need for improved preclinical models of human reproductive immunology. Yet, upon depletion of T_REGs_, CD8 T cells can home to uterine tissues of pregnant dams, increasing resorptions, whereas adoptive transfer of pregnancy-induced T_REGs_ prevents resorptions (6, 25). Similarly, viral infection during murine gestation allows recruitment of CD8 T cells to uterine and placental tissues, yet without effective clearance of placental infection (26).

Due to the importance of pregnancy for species survival, it is expected that a multitude of mechanisms contribute to reducing the risk of maternal T cell rejection of fetal and placental allo-antigens, including select expression of MHC by fetal trophoblasts to limit NK cell and T cell cytotoxicity (1, 18), select expression of chemokines to limit infiltration of pathogenic T cells (24), activation and induction of T_REGs_ with specificity for fetal antigens (27–29), and peripheral deletion of fetal-reactive T cells in mice (30), but not in humans (15, 16). While these mechanisms of tolerance likely inhibit the effective clearance of infections from uterine tissues in pregnant mice and humans (26, 31), not all mechanisms of immunity are suppressed, as most pregnant people can mount effective immune responses to vaccines and pathogens that protect against congenital infections (32–34).

The constraints of human research and the lack of affordable animal models with T cell diversity reflecting human MFI tissues have hindered advancement in our understanding of T cell immunology at the MFI (1, 3, 18, 24, 35). We and others have demonstrated that the practice of raising mice in conventional SPF conditions stunts immune development relative to humans and wild mice, particularly the formation of a robust memory T cell compartment in tissues (36–42). Raising mice in diverse microbial environments does not negatively impact the health and survival of offspring provided the mother is acclimated to the environment; in fact, microbially exposed offspring demonstrate improved immune development and host defense relative to SPF pups (36).

Therefore, we sought to determine whether the paucity of T cells in SPF mouse MFI results from the lack of microbial exposure. To this end, we employed a natural microbial exposure (NME) model whereby laboratory mice are co-housed with pet store mice to passively share their diverse microbial community prior to breeding. We found, maternal microbial experience expanded T cell populations and increased T cell diversity at the MFI relative to SPF dams in a manner that closely phenocopied our observations in humans, displaying resident memory and mixed activation/dysfunction gene expression signatures. The experiments described here amend our understanding of the immune landscape at the MFI, underscoring the influence of maternal microbial experience. In a significant step forward for the field we have established an improved translational model of human pregnancy that permits mechanistic study of the immunology of the MFI.

## Materials and methods

### Mice

Male and female C57BL/6J (B6) were purchased from Jackson Laboratories (Bar Harbor, ME). Female pet store mice were purchased from local pet stores in the Minneapolis-Saint Paul, Minnesota metro area. To naturalize female B6 mice we cohoused them with pet store mice for >4 wks. During proestrus, naturalized B6 female mice are transferred to a cage housing a male B6 mouse for timed overnight mating. Successful mating is confirmed by the presence of a copulatory plug. The female B6 mouse is then returned to the pet store cage where she will remain for the duration of the experiment. Serological testing has revealed pet-store mice often carry some combination of rotavirus (epizootic diarrhea of infant mice), mouse hepatitis virus, murine norovirus, mouse parvovirus NS1, type 1/2, minute virus of mice, Theiler’s murine encephalomyelitis virus (TMEV), Sendai virus, lymphocytic choriomeningitis, mouse adenovirus types 1 and 2, mouse CMV, polyomavirus, pneumonia virus of mice, Mycoplasma pulmonis, Clostridium piliforme, pinworms, fur mites, and Encephalitozoon cuniculi. Pet-store mice were also tested for ectromelia virus (mousepox), reovirus, and cilia-associated respiratory Bacillus, but have not tested positive for these microbes. Our specific pathogen free (SPF) colony is routinely tested to ensure the absence of the following pathogens: mouse parvovirus, minute virus of mice, mouse hepatitis virus, mouse rotavirus-A (epizootic diarrhea of infant mice), Theiler’s murine encephalomyelitis virus (TMEV), Sendai virus, pneumonia virus of mice, reovirus, Ectromelia (Mousepox), mouse adenovirus types 1 and 2, polyomavirus, lymphocytic choriomeningitis virus, mouse CMV, Mycoplasma pulmonis, Clostridium piliforme (Tyzzer’s disease), cilia-associated respiratory Bacillus, fur mites (Myobia musculi, Radfordia affinis, Radfordia ensifera, Myocoptes musculinus), pinworms (Aspiculuris tetraptera, Syphacia obvelata, Syphacia muris), and Encephalitozoon cuniculi. Mice were sacrificed at 14.5 dg for experiments. All mice were housed in Association for Assessment and Accreditation of Laboratory Animal Care-approved animal facilities at the University of Minnesota (BSL-1 for SPF mice and BSL-3 for cohoused mice). All animal use was performed per a University of Minnesota Institutional Animal Care and Use Committee approved protocol (2404-42036A).

### Human placental tissue collection and processing

Placental tissues and maternal blood samples from full-term pregnancies (gestational age >39 weeks) were collected from healthy women with uncomplicated pregnancies that delivered by elective aesarean sections under IRB approved protocols. To further ensure quality, the tissues were inspected for signs of placental inflammation (such as discoloration, significant infarctions, or bad odor), and only healthy tissue samples were selected for further use. Maternal blood samples were collected directly before delivery. Detailed procedures to isolate decidual, villous, and peripheral blood CD8+ T cells were recently described (21, 43). Briefly, decidua basalis tissue was dissected from the maternal side of the placenta and villi were removed from the decidua basalis. Villous tissue was collected separately to purify placental immune populations as described (43, 44). Collected decidual and villous tissues were washed extensively and then minced and digested with 0.1% collagenase type IV and 0.01% Dnase I (Sigma-Aldrich) for 75 minutes at 37°C in a gently shaking water bath. Following digestion, the cells were washed and filtered through 100-, 70-, and 40-μm cell strainers (BD, Labware). Lymphocytes were suspended in 20 mL of 1.023 g/mL Percoll (GE Healthcare) and subjected to a gradient centrifugation, layered with 10 mL of 1.080 g/mL and 15 mL of 1.053 g/mL Percoll. After 30 minutes of centrifugation at 800g, lymphocytes were harvested from the interface of the 1.080 and 1.053 g/mL layers and subsequently washed. Peripheral CD8+ T cells were isolated using RosetteSep (StemCell Technologies) and a Ficoll gradient (GE Healthcare) during a 20-minute centrifugation at 800g. Finally, the blood and lymphocyte samples were washed and stained for flow cytometry using a Cytek Aurora spectral flow cytometer.

### IV labeling and tissue harvest

Mice were sedated with isoflurane prior to retroorbital intravenous (IV) injection of 5 μg of anti-CD45.2-APC (Proteintech) antibody to label leukocytes in contact with the circulation. Mice were then sacrificed after 3 min for tissue harvest. Concepti were removed and placenta and decidua were dissected. Tissue was mechanically separated and filtered through a 70 μm filter to form a single cell suspension. Cells are then stained with anti-CD45 conjugated to a different fluorophore (i.e. BV605), which binds surface CD45 on leukocytes not already occupied by the IV injected anti-CD45-APC. This method permits the delineation of leukocytes in circulation from those within tissue.

### Flow cytometry

Mouse cells were washed with FACS buffer and stained for flow cytometry using antibodies referenced in **Table 1**. CD1d tetramer was prepared by incubating PBS-57-loaded CD1d biotinylated monomer (NIH Tetramer Core Facility) with Streptavidin PE-Cyanine7 conjugate (eBioscience, catalog number 25-4317-82). After extracellular staining, cells were washed, fixed, and permeabilized (BD Biosciences). Permeabilized cells were intracellularly stained using the antibodies referenced in **Table 1**. Cells were washed and flow cytometric analysis was performed on the Cytek Aurora (Cytek Biosciences). Data were analyzed with FlowJo Software version 10 (BD Biosciences). Surface staining of human cells was performed by incubating cells for 30 minutes in RPMI medium supplemented with penicillin/streptomycin and 10% newborn calf serum. For intracellular staining, cells were fixed and permeabilized using the CytoFix/CytoPerm kit (BD). Data acquisition was performed on a Cytek Aurora, and data were analyzed using FlowJo software (BD Biosciences).

**Table 1:** Antibodies used for flow cytometric and immunohistochemical analyses.

Human Antibodies					
Target	Clone	Fluorophore	Dilution	Company	Catalog number
CCR7	G043H7	Alexa488	1:50	BioLegend	353206
CD3	UCHT1	BUV737-Tdm	1:100	BD Biosciences	612750
CD4	M-T477	BUV395-Tdm	1:100	BD Biosciences	742738
CD8	RPA-T8	BUV805-Tdm	1:100	BD Biosciences	749366
CD14	63D3	BV510	1:50	BioLegend	367124
CD16	B73.1	BV480	1:50	BD Biosciences	746514
CD19	SJJJ25C1	BUV661-Tdm	1:100	BD Biosciences	750536
CD25	2A3	PE	1:50	BD Biosciences	341010
CD39	A1	PE/Dzle594	1:50	BioLegend	328224
CD45	2D1	SB550	1:50	BioLegend	368549
CD45RA	HI100	PE/Cy5	1:100	BioLegend	304110
CD56	NCAM16.2	BUV496-Tdm	1:100	BD Biosciences	750479
CD69	FN50	BV605-Tdm	1:50	BioLegend	310938
CD103	Ber-ACT8	BV421	1:50	BioLegend	350214
FOXP3*	259D	P. Blue	1:50	BioLegend	320216
Granzyme B*	QA16A02	APC/Fire750	1:50	BioLegend	372210
PD1	EH12.1	PE/Cy7	1:100	BD Biosciences	561272
Perforin*	dG9	BV711-Tdm	1:50	BioLegend	308130
CCR7	G043H7	Alexa488	1:50	BioLegend	353206
CD3	UCHT1	BUV737-Tdm	1:100	BD Biosciences	612750
CD4	M-T477	BUV395-Tdm	1:100	BD Biosciences	742738
Mouse Antibodies					
**Target**	**Clone**	**Fluorophore**	**Dilution**	**Company**	**Catalog Number**
CD3	145-2C11	BUV395	1:50	BD Biosciences	563565
Ly6G	1A8	BUV395	1:100	BD Biosciences	563978
CD4	GK1.5	BUV496	1:50	BD Biosciences	612952
CD69	H1.2F3	BUV563	1:50	BD Biosciences	741234
NK1.1	PK136	BUV563	1:50	BD Biosciences	741233
CD49a	Ha31/8	BUV661	1:50	BD Biosciences	741549
CD44	IM7	BUV737	1:100	BD Biosciences	612799
CD8	53-6.7	BUV805	1:50	BD Biosciences	612898
CD103	2E7	BV421	1:20	BioLegend	121422
TCRgd	GL3	BV421	1:100	BioLegend	118120
PD-1/CD279	J43	BV480	1:20	BD Biosciences	746784
CD44	IM7	V500	1:50	eBioscience	12-0441-82
Live/Dead	N/A	BV510	1:100	Tonobo	13-0870-T100
CD45.2	104	BV570	1:20	BioLegend	109833
CD19	6D5	BV605	1:40	BioLegend	115539
CD45.2	104	BV605	1:50	BioLegend	109841
TCRb	H57-597	BV605	1:100	BD Biosciences	562840
CD69	H1.2F3	BV650	1:100	BioLegend	104530
Ly6C	HK1.4	BV650	1:80	BioLegend	128049
RORγt*	Q31-378	BV785	1:40	BD Biosciences	564723
CD103	M290	BV786	1:50	BD Biosciences	564322
CD45.2	104	AF488	1:20	BioLegend	109816
CD11c	N418	FITC	1:100	BioLegend	117305
CD62L	MEL-14	BB515	1:50	BD Biosciences	565261
B220	RA3-6B2	PerCP-Cy5.5	1:100	BioLegend	103236
CD44	IM7	PerCP-Cy5.5	1:40	eBioscience	45-0441-82
FOXP3*	FJK-16s	PerCP-Cy5.5	1:150	eBioscience	45-5773-82
CD25	PC61.5	PerCP-Cy5.5	1:135	eBioscience,	45-0251-80
Granzyme B*	QA16A02	PE	1:20	BioLegend	372208
Perforin*	S16009A	PE-Dazzle594	1:75	BioLegend	154316
CD39	Duha59	PE-Fire640	1:200	BioLegend	143818
CD1d tetramer	N/A	PE-Cy7	1:65	N/A	N/A
CD45.2 (IV)	104	APC	N/A	Proteintech	65072
NK1.1	PK136	APC	1:50	BD Biosciences	550627
PD-1	29F.1A12	APC	1:20	BioLegend	135210
B220	RA3-6B2	APC-efluor780	1:100	eBioscience	47-0452-82
CD11c	N418	APC-efluor780	1:100	eBioscience	47-0114-82
CD11b	M1/70	APC-efluor780	1:100	eBioscience	47-0112-82
NK1.1	PK136	APC-efluor780	1:100	eBioscience	47-5941-82
TER-119	TER-119	APC-efluor780	1:100	eBioscience	47-5921-82
F4/80	BM8	APC-efluor780	1:100	eBioscience	47-4801-82

*Denotes antibodies included in intracellular stain.

### Histology and immunohistochemistry

Concepti, including the placenta and decidua, were placed in OCT compound and frozen at -80°C. Tissue blocks were then sectioned with a cryostat and prepared for slides. Serial sections were stained with hematoxylin and eosin (Vector Labs) or with AF488 anti-CD144/VE-cadherin (Thermo Fisher, reference # 53-1441-832), AF647 anti-CD324/E-cadherin (Biolegend, catalog # 147308), and Vectashield with DAPI (Vector Labs) and imaged for immunofluorescence. Slides were imaged with a Leica DM6000 Thunder Epifluorescent Microscope equipped with a K8 CCD camera or Leica SP8 inverted confocal/epifluorescence scope equipped with a Leica DFC7000 T Camera. Images were analyzed with Leica X software.

### Single cell sequencing

MFI (placenta and decidua combined) from CD45.2 IV-labeled SPF and NME mice were isolated at 14.5 dg. Cells were stained with the antibodies referenced in **Table 1**. Live CD45+ leukocytes were analyzed by fluorescence-activated cell sorting (FACS) and sorted into IV+ and IV- populations. Cell captures and 5’ gene expression libraries were prepared using the 10X Chromium X instrument (10X Genomics) with the assistance of the University of Minnesota Genomics Core (UMGC). Samples underwent quality control sequencing on the MiSeq QC (Illumina) followed by full sequencing with the NovaSeq X Plus (Illumina). Samples were analyzed in R using Seurat V5 using standard methods and cell clusters were annotated with the help of scType cell-type identification package (45, 46).

### Quantification and statistical analysis

Data were collected across multiple experiments performed over 2 years. GraphPad Prism 9 was used to perform statistical analysis. A two-tailed unpaired, nonparametric Mann-Whitney U test was used to compare two groups at a single time point. Where three groups were compared Kruskal-Wallis test was used with Dunn’s multiple comparisons test.

## Results

### Maternal microbial exposure is compatible with healthy pregnancy

**Figure 1:**
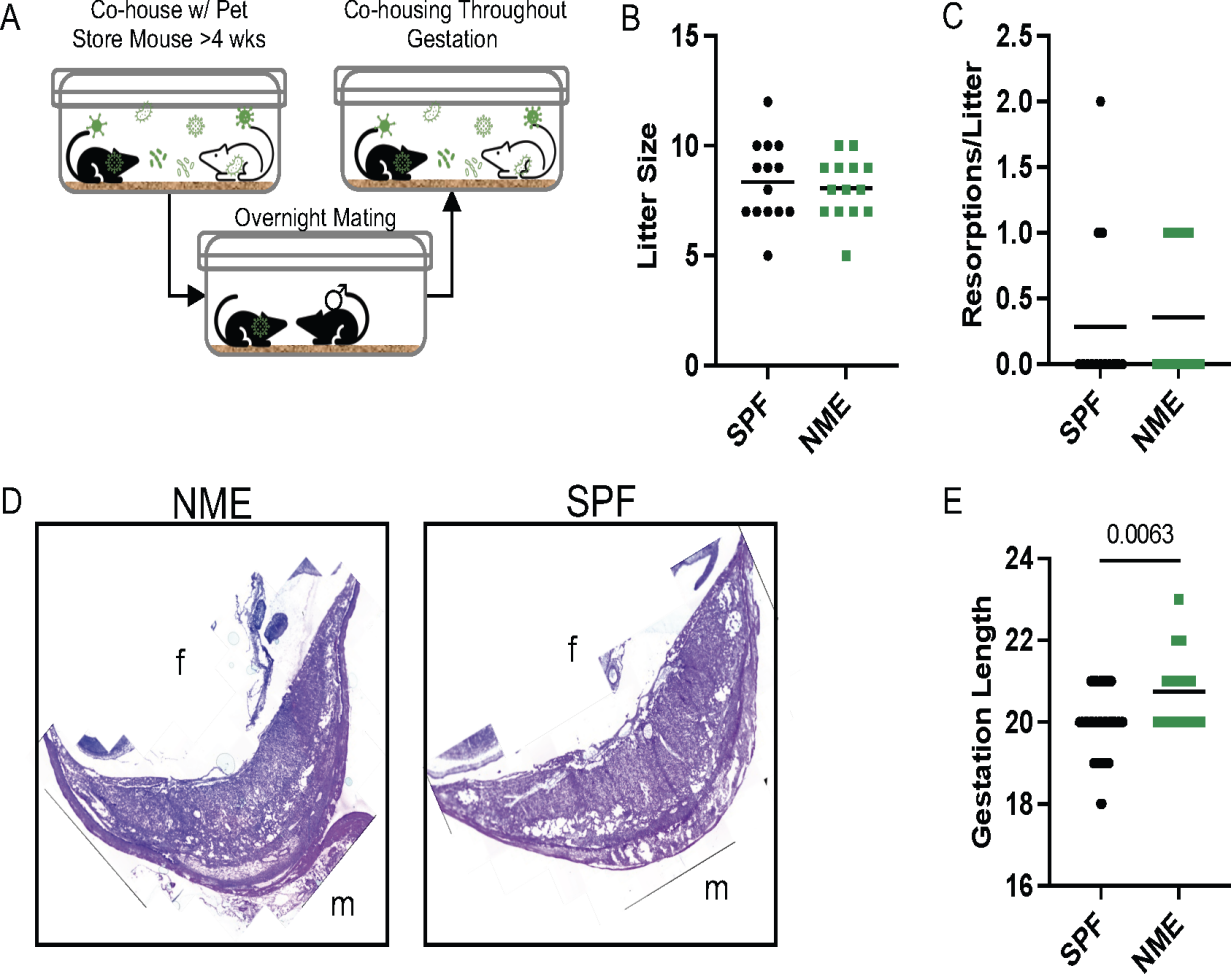
NME model of reproductive immunology. **(A)** Female C57BL/6 mice are cohoused with pet store mice for >4 weeks, referred to as natural microbial exposure (NME). Female NME mice are placed into a separate cage with a male C57BL/6 mouse during proestrus for overnight breeding. The following morning the NME female mouse is returned to her co-housing cage, where she will remain for the duration of the experiment. Pregnant NME and SPF female mice were euthanized at 14.5 dg. We observed **(B)** litter size and **(C)** concepti resorptions (n = 14 litters per group). **(D)** The maternal fetal interface (MFI) was flash frozen in OCT and sectioned for hematoxylin and eosin (H&E) staining. Images are labelled with maternal (m) and fetal (fetal) orientation. **(E)** Average length of gestation under SPF and NME conditions (n = 16 litters for NME and 51 litters for SPF). Mann-Whitney U test was used to determine significance.

To investigate how maternal exposure to diverse microbial communities influence the immune populations at the MFI, we cohoused female C57BL/6 mice with female pet store mice for a minimum of 4 wks to passively share their diverse microbial communities. After this acclimation period, female C57BL/6 mice were transferred during proestrus to a separate cage with a male C57BL/6 mouse for timed overnight mating. The following morning, female mice were checked for a copulatory plug and returned to their cohousing cage where they remained for the duration of the experiment (**Figure 1A**) (36). This ensures the maternal immune system and other maternal factors influenced by microbial experience are normalized prior to conception and throughout gestation. NME did not impact the average size of the litter or the number of resorptions per litter (**Figures 1B, C**), confirming previous observations (36). In addition, there were no gross histologic differences between NME and SPF placenta and decidua (**Figure 1D**; **Supplementary Figure S1**). Interestingly, NME-exposed mice had a slight but significantly increased length of gestation compared to conventionally housed SPF mice (**Figure 1E**). Thus, cohousing female C57BL/6 mice with female pet store mice is an innovative pregnancy model compatible with healthy murine pregnancy and pup survival.

### Maternal microbial experience alters leukocyte populations at the MFI

To determine how maternal microbial exposure alters immune cells at the MFI, we performed single cell RNA sequencing (scRNAseq) on CD45.2+ cells purified from the placental tissues of SPF and NME dams at 14.5 days of gestation (dg). The tissue included both decidual and villous tissues to capture all MFI immune cell types. In addition, IV labeling with anti-CD45.2-APC was performed to purify IV+ circulating and IV– resident leukocytes by FACS sort before making cDNA libraries (see *Materials and Methods*). Interestingly, ~80% of decidual immune cells (~90% of T cells) were IV–, indicating these consist of predominant tissue resident immune populations. In contrast, and likely due to the vascular nature of the placenta, a high level of IV+ staining (~80% of immune cells and ~90% of T cells) was found in the placental tissue (**Supplementary Figures S2A–D**). NME conditions did not appear to alter the proportions of IV labeled cells (**Supplementary Figures S2B, D**). Thus, IV labeling may serve as a proxy to separate decidual and placental immune cells in the scRNA-seq data.

**Figure 2:**
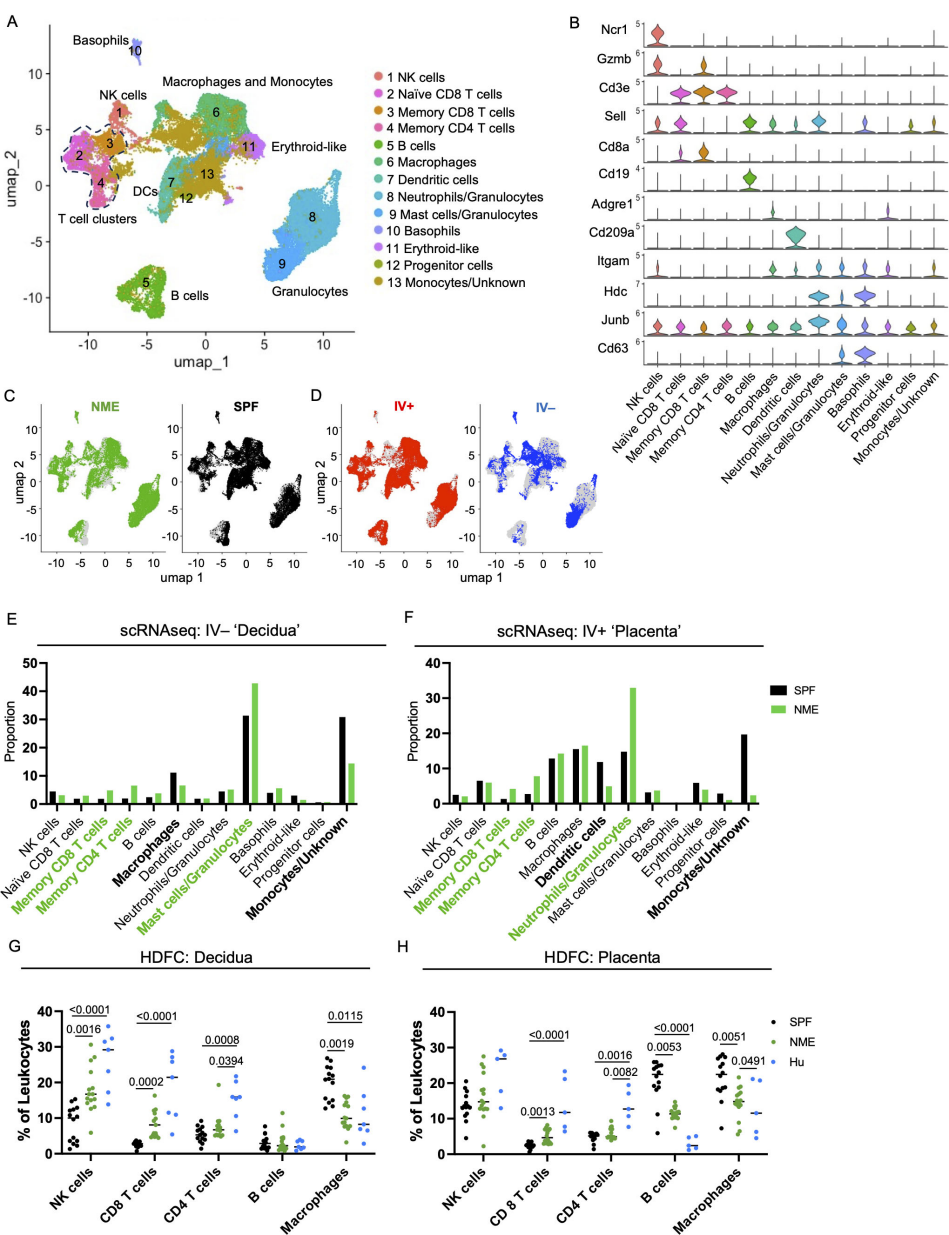
NME alters immune cell composition at the MFI. IV-labeled CD45+ immune cells were sorted from the MFI of NME and SPF dams at 14.5 dg and used for single cell transcriptomic analysis (scRNAseq) and high dimensional flow cytometry (HDFC) (n = 3 litters/group). **(A)** We performed dimensional reduction of the scRNAseq dataset using uniform manifold approximation and projection (UMAP) in Seurat and the scType cell identification R package to identify 13 immune cell clusters. T cell clusters are outlined with dotted line. **(B)** Key markers were used to confirm and refine cluster annotation. The location of **(C)** NME (green) and SPF (black) and **(D)** IV-labeled immune cells (red IV+ and blue IV negative) on the UMAP plot revealed cluster biases. Immune cell composition of **(E)** decidua and **(F)** placenta in NME (green) and SPF (black) mice determined by scRNAseq. Cell populations enriched in the NME and SPF samples are in bolded green and black, respectively. HDFC of immune cell composition in the **(G)** decidua and **(H)** placenta of NME mouse (green), SPF mouse (black), and human (blue) samples (n = 14–15 MFI from 4–5 separate litters, n = 8 for human samples). Mann-Whitney U test was used to determine significance.

Uniform manifold approximation and projection (UMAP) graphs and the ScType cell classification package in R were used to help identify and annotate 13 immune cell clusters (**Figure 2A**) (46). Immune cell cluster identities were confirmed by expression of several key lineage markers (**Figure 2B**; **Supplementary Figure S3A**). SPF and NME mice tissues contained immune cells spanning all clusters (**Figures 2C, D**) but had marked differences. Using IV labeling to enrich for placental and decidual immune cells, we found that NME i) increased frequencies of several memory CD8 and CD4 T cell types in both placental and decidual tissues; ii) increased frequencies of neutrophils/granulocytes in the placenta and mast cells/granulocytes in the decidua; iii) decreased frequencies of macrophages in the decidua; and iv) decreased frequencies of dendritic cells in the placenta (**Figures 2E, F**). The transcriptomic signature of NME MFI macrophages showed an increase in expression of pro-inflammatory genes associated with M1 polarization (i.e., *Socs3*, *FosB*, *Jun*, *Hspa1a*, and *Il1b*), as well as an increase in expression of the anti-inflammatory/M2-associated *Dusp1* gene relative to SPF dams (**Supplementary Figure S4A**). Interestingly, while the frequencies of NK and B cells in the MFI were largely the same in SPF and NME mice, NK and B cells from the MFI of NME dams were also marked by an increase in the expression of *Jun*, suggesting enhanced activation in NME mice (**Supplementary Figures S4B, C**). Thus, maternal microbial experience induces significant changes in decidual, and placental leukocyte composition and activation states compared to SPF housed pregnant mice.

### Leukocyte composition in MFI of NME mice reflect human MFI leukocytes

Next, we performed high dimensional flow cytometry (HDFC) on mechanically separated placental and decidual tissues harvested from SPF and NME dams at 14.5 dg to enumerate immune cells, confirm the scRNAseq data, and compare immune populations with human decidual and placental tissues (**Figures 2G, H**, **Supplementary Figures S3A–C**). In agreement with the scRNAseq data, HDFC demonstrated decidual and placental T cells were expanded in NME dams relative to SPF dams (**Figures 2G, H**). Specifically, CD8 T cells were virtually absent from MFI tissues in SPF dams and present in MFI tissues of NME dams in a range that more closely resembles the CD8 T cell frequencies found in human decidual and placental tissues (**Figure 2G, H**). In addition, a marked increase in the frequency of decidual NK cells was observed in NME dams relative to SPF dams, with a range that resembled the decidual NK cell frequency in human tissues with improved fidelity. Confirming the scRNAseq data, a decrease in the frequency of decidual and placental macrophages in NME dams relative to SPF dams was observed (**Figures 2G, H**). A comparison with human macrophage and B cell frequencies again shows that the range of frequencies observed in NME dams resemble human MFI macrophage frequencies with greater fidelity than those observed in SPF dams. CD8 T cells were also found to be more numerous and macrophages less numerous in the decidua and placenta, while B cells were less numerous in the placenta of NME dams relative to SPF dams, demonstrating NME induces both a change in frequency and absolute number for these populations (**Supplementary Figures 3B, C**). Altogether, these data demonstrate maternal microbial exposure alters the immune cell composition of the murine MFI and phenocopy the human MFI immune cell populations with great fidelity.

### Maternal microbial experience influences T cell composition at the MFI

**Figure 3:**
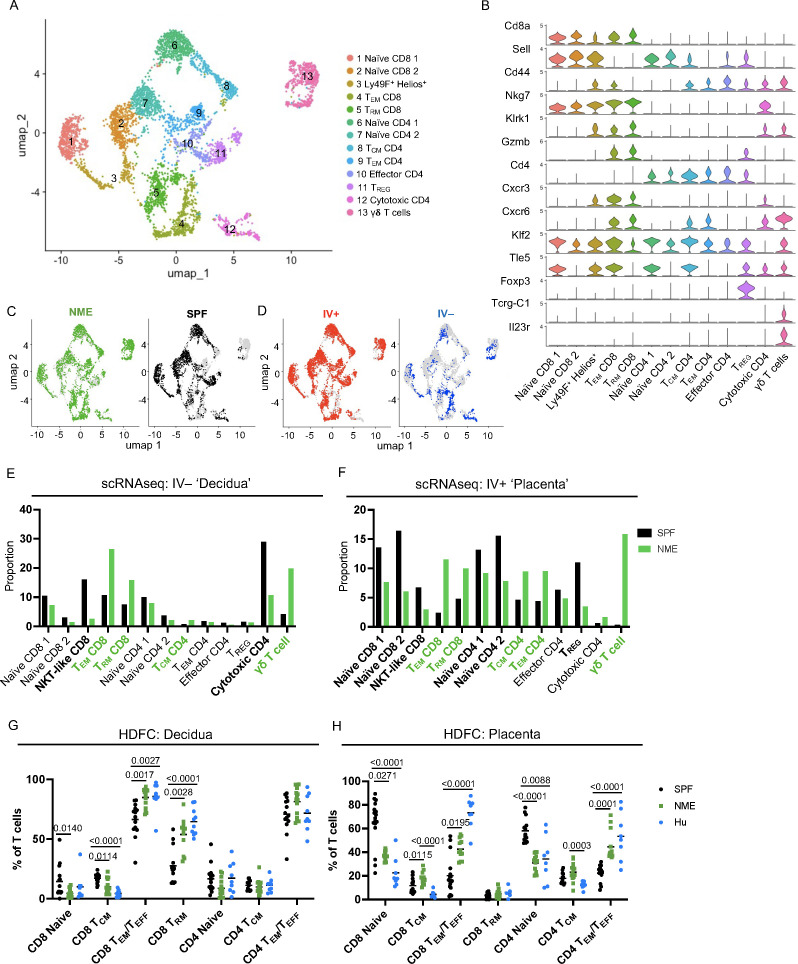
NME alters T cell composition at the MFI. **(A)** T cells were sub-gated from the scRNAseq dataset and UMAP plots were made and annotated as before. **(B)** Key markers were used to confirm and refine cluster annotation. **(C)** NME (green) and SPF (black) and **(D)** IV-labeled cells (red IV+ and blue IV negative) were identified on the UMAP plot. scRNAseq data revealed NME conditions skewed the T cell population toward activated subsets and away from naïve subsets in both the **(E)** decidua (IV–) and **(F)** placenta (IV+). T cell composition of **(E)** decidua and **(F)** placenta in NME (green) and SPF (black) mice determined by scRNAseq. T cell populations enriched in the NME and SPF samples are in bolded green and black, respectively. HDFC of T cell populations in the **(G)** decidua and **(H)** placenta of NME mouse (green), SPF mouse (black), and human (blue) samples. Sample size is 14–15 MFI from 4–5 separate litters. Sample size is 8 for human samples. Mann-Whitney U test was used to determine significance.

CD4 and CD8 memory T cell populations are dominant immune populations in human MFI tissues at term pregnancy (17–23). Consequently, the significant expansion of T cells under NME conditions compared to SPF conditions has high translational significance. To provide further resolution of how NME changes T cell diversity at the MFI, scRNAseq resolved T cell clusters were selected for further analysis. UMAPs of all T cells from SPF and NME MFI tissues identified 13 separate T cell clusters (**Figure 3A**). T cell cluster identities were confirmed by expression of key markers (**Figures 3A, B**; **Supplementary Figure 6**). Stark differences in the distribution of T cells across NME and SPF dams as well as decidual and placental tissues were observed. Overall, a strong bias towards activated T cells (clusters 4, 5, 8, 9, and 13) in the NME tissues was found (**Figures 3C, D**). Of the IV– decidual T cells in NME samples, a greater proportion of i) CD8 T_EM_ and T_RM_ cells (clusters 4 and 5); ii) γδ T cells (cluster 13); iii) CD4 T_CM_ cells (cluster 8) were found; combined with iv) lower frequencies of *Klra6* (Ly49F)+ *Ikzf2* (Helios)+ T cells (cluster 3) and cytotoxic CD4 cells (cluster 12) were observed (**Figure 3E**). NME IV+ placental T cells demonstrated an even greater skewing toward memory T cell populations, with increased proportions of CD4 and CD8 memory T cells (clusters 4, 5, 8, and 9) and γδ T cells (cluster 13) (**Figure 3F**). While proportions of naïve T cells (clusters 1, 2, 6, and 7) and *Klra6* (Ly49F)+ *Ikzf2* (Helios)+ T cells (cluster 3) were decreased in NME mice relative to SPF mice (**Figure 3F**). These observations were confirmed by HDFC where we also observed increased proportions of memory T cell subsets in both the decidua and placenta and corresponding decreases in naïve T cell populations in the placenta (**Figures 3G, H**). Direct comparison of SPF, NME, and human T cell diversity by HDFC showed NME conditions replicated human conditions more accurately than SPF conditions. Specifically, the increased proportion of CD8 T_EM_/T_EFF_ and T_RM_ subsets in the decidua and placenta and the decrease of CD4 and CD8 naïve T cells in both tissues of NME mice match human T cell diversity in more detail (**Figures 3G, H**). CD4 and CD8 T_EM_/T_EFF_ cells were also more numerous in both decidua and placenta, and CD4 and CD8 T_CM_ cells were more numerous in the placenta of NME dams than SPF dams (**Supplementary Figures 6B, C**). Thus, maternal microbial exposure induces profound changes in the composition of T cells at the MFI, with stark increases in tissue memory CD8, CD4, and γδ T cell types. These data establish NME mice as an improved translational model for human immunology at the MFI.

### Maternal microbial experience increases CD8 T cell activation and diversity at the MFI

**Figure 4:**
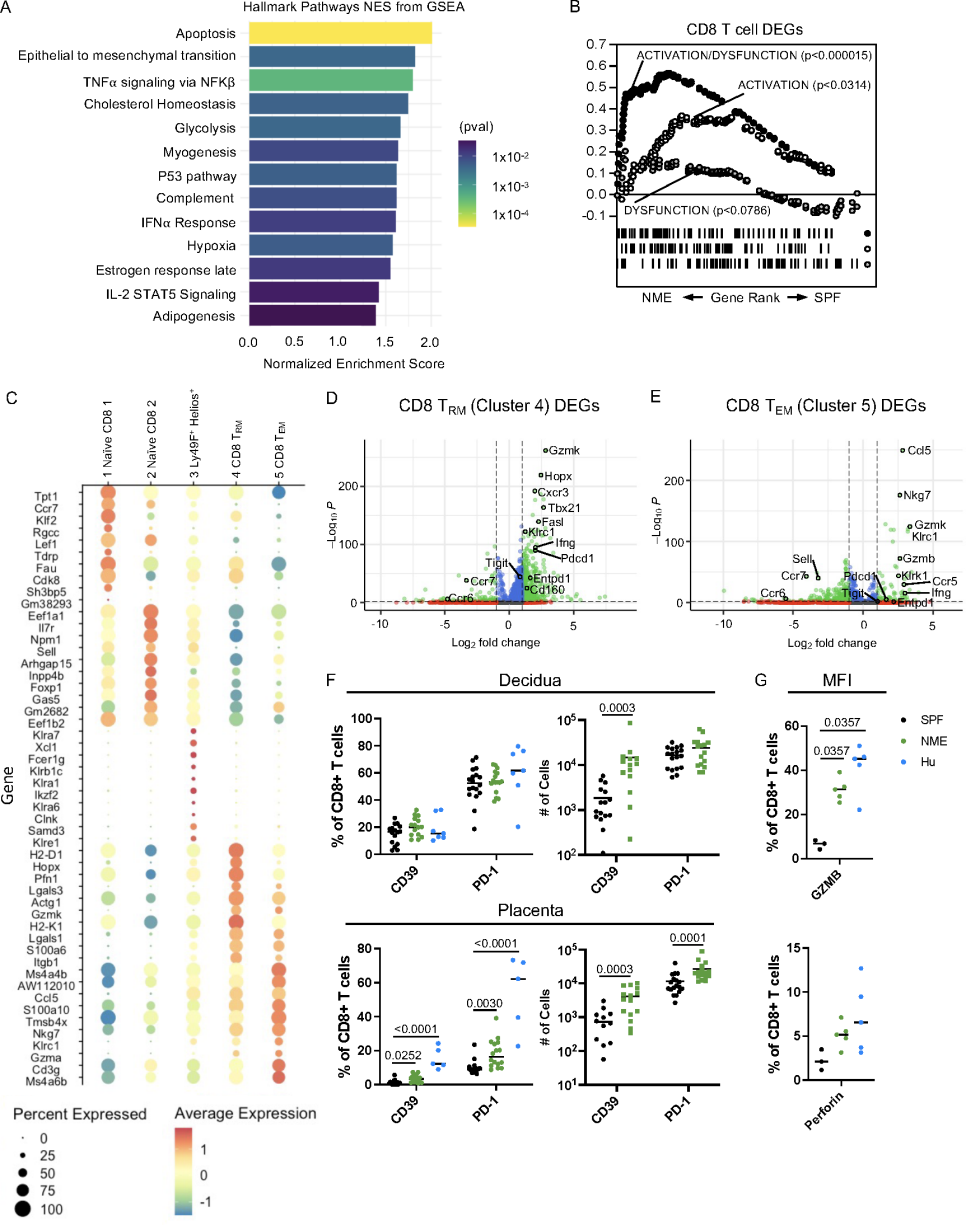
NME MFI CD8 T cells phenocopy those of humans. **(A)** GSEA was used to identified Hallmark Pathways associated with NME CD8 T cells. **(B)** Gene sets for activation/dysfunction, activation, and dysfunction were compared to DEGs from NME CD8 T cells using gene rank. **(C)** The top ten DEGs defining each CD8 T cell cluster were determined using Seurat. Volcano plots showing DEGs from **(D)** CD8 T_RM_ (cluster 4) and **(E)** CD8 T_EM_ (cluster 5). **(F)** Proportion and number of CD39 and PD-1 expressing CD8 T cells in the decidua (top) and placenta (bottom) determined flow cytometrically of NME mouse (green), SPF mouse (black), and human (blue) samples ((n = 15–18 MFI from 4–5 separate litters, 7 for human samples). **(G)** Granzyme B (left) and Perforin (right) protein expression in T cells harvested from the MFI (decidua and placenta combined) of NME mouse (green), SPF mouse (black), and human (blue) samples (n = 3 for SPF, 5 for NME, and 5 for human). Mann-Whitney U test was used to determine significance.

To further define functional changes in NME-expanded CD8 T cell clusters, differential gene expression analysis between total NME and SPF MFI CD8 T cells was performed using Seurat. Gene Set Enrichment Analysis (GSEA) found several hallmark pathways including “TNFα-signaling via NFKβ“, “Complement”, “IFNα Response”, and “IL-2 STAT5 Signaling” significantly enriched in CD8 T cells of NME MFI, a finding in line with the expectation of increased basal immune activation in NME mice (**Figure 4A**). Hallmark pathways in NME CD8 T cells further indicated i) a marked increase in apoptosis, glycolysis, cholesterol, adipogenesis, and hypoxia pathways, suggesting a skewing of CD8 T cell viability and metabolic pathways; and ii) an increase in late estrogen response genes, suggesting that pregnancy hormones have a stronger influence on CD8 T cell activation and differentiation in NME compared to SPF dams (**Figure 4A**). To further define how the activation state of the NME expanded MFI CD8 T cells compare to human MFI T cells, we applied GSEA using curated datasets of ‘Activation’, ‘Dysfunction’, and ‘Activation/Dysfunction’ as we described previously (21, 47). Similar to what we demonstrated in humans, MFI CD8 T cells from NME dams overexpressed a mixed signature of ‘Activation/Dysfunction’ as compared to SPF mice, further confirming the greater similarity of MFI immunology between NME mice and humans (**Figure 4B**) (21, 47).

Differentially expressed genes (DEGs) from CD8 T_Rm_ and T_em_ cells (clusters 4 and 5), which were enriched in NME dams relative to SPF dams, revealed increased expression of multiple markers associated with cytotoxicity including *Nkg7*, *Gzmk*, *Gzma*, *Gzmb, and Ifng* as well as markers associated with regulation (*Pdcd1*, *Cd160*, and *Entpd1*) (**Figures 4C–E**). In agreement with the transcriptomic data, a greater proportion and number of CD8 T cells expressing PD-1 or CD39 protein in the placenta of NME dams relative to SPF dams was detected by HDFC (**Figure 4F**). Likewise, intracellular expression of granzyme B and perforin protein by CD8 T cells at the MFI (combined decidua and placenta) was higher in NME dams relative to SPF dams (**Figure 4G**). Both an increase in markers of CD8 dysfunction (PD-1 and CD39) and in markers of activation (GZMB and PFN) align the CD8 T cell phenotype of NME dams more closely with human MFI CD8 T cells (21, 22).

### Maternal microbial experience increases CD8 T cell residency at the MFI

**Figure 5:**
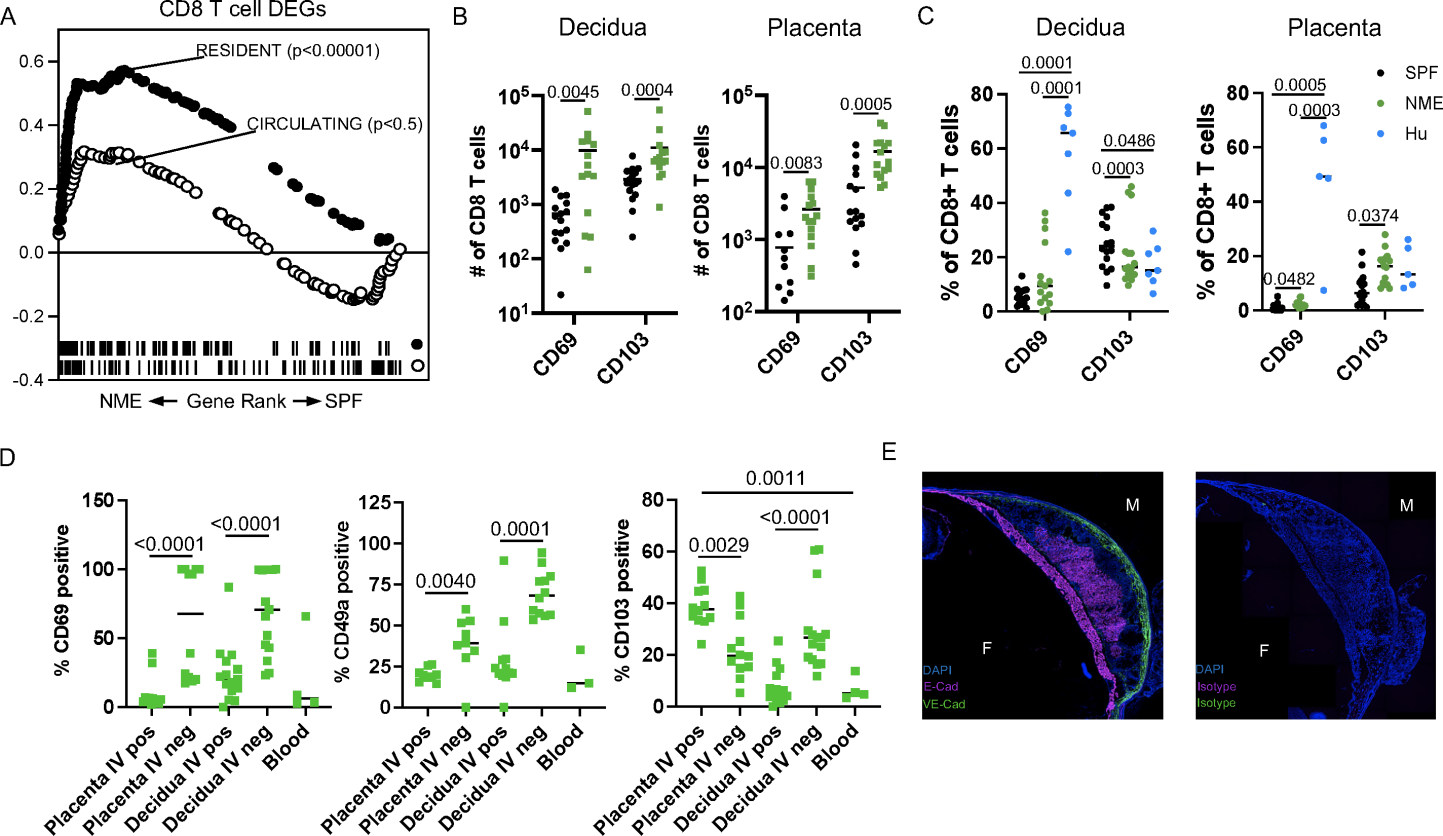
NME induces the expansion of cells expressing a residency phenotype. **(A)** GSEA using gene sets for resident and circulating cells was performed on NME differentially expressed genes (DEGs) from MFI CD8 T cells. **(B, C)** Number **(B)** and proportion **(C)** of CD69 and CD103 expressing CD8 T cells from SPF (black), NME (green) and human (blue) decidua (left) and placentas (right). Sample size is 14–15 MFI from 4–5 separate litters. **(D)** Flow cytometric measurement of common markers of T cell residency (CD69, CD49a, and CD103) in CD45.2 IV+ and IV- NME CD8 T cells from decidua and placenta compared to maternal blood (n = 9–12 from 4 separate litters). **(E)** Immunohistochemistry staining of CD103 ligand, E-cadherin, VE-cadherin, or isotype expression with DAPI from NME frozen MFI sections.

Besides the strong increase in CD8 activation and dysfunction, our prior studies of human MFI CD8 T cells demonstrated a predisposition towards the tissue residency phenotype (18, 19, 22). Using GSEA and published gene sets of ‘Resident’ and ‘Circulating’ T cells curated by Milner et al., we expectedly found that the NME CD8 T cell DEGs strongly aligned with the ‘Resident’ gene set (**Figure 5A**) (48). We measured the expression of commonly used markers of residency (CD69 and CD103) in decidual and placental CD44+ CD62L– effector CD8 T cells and observed an increased in the number of these cells in NME tissues relative to SPF tissues (**Figure 5B**), that more closely aligned with human CD8 T cells (**Figure 5C**). In mice, we found CD69 and CD49a expression were more associated with IV– cells than IV+ CD8 T cells in the decidua and placenta in agreement with these being markers of residency (**Figure 5D**). However, in the placenta, a greater proportion of IV+ cells were CD103+ than IV– cells, while IV– cells were more likely to be CD103+ than IV+ cells in the decidua (**Figure 5D**). CD103 expression was very rare in CD44+ effector/memory CD8 T cells in the peripheral blood, demonstrating placental IV+ T cells are unique from circulating T cells and may contain ‘resident’ T cells.

Immunofluorescence staining confirmed the expression of the CD103 ligand, E-cadherin, in the chorionic plate and placental labyrinth of both NME and SPF mice, similar to humans, suggesting CD103 expression may help recruit and/or retain T cells at the MFI (**Figure 5E**) (49). Altogether, these data demonstrate that NME expands T cell subsets with a resident memory-like phenotype in both placental and decidual tissues. These data also demonstrate that placental T cells, despite IV labeling, are unique from peripheral blood and express receptors for placental tissue ligands that may promote recruitment and residency.

### Maternal microbial experience increases CD4 T cell diversity at the MFI

**Figure 6:**
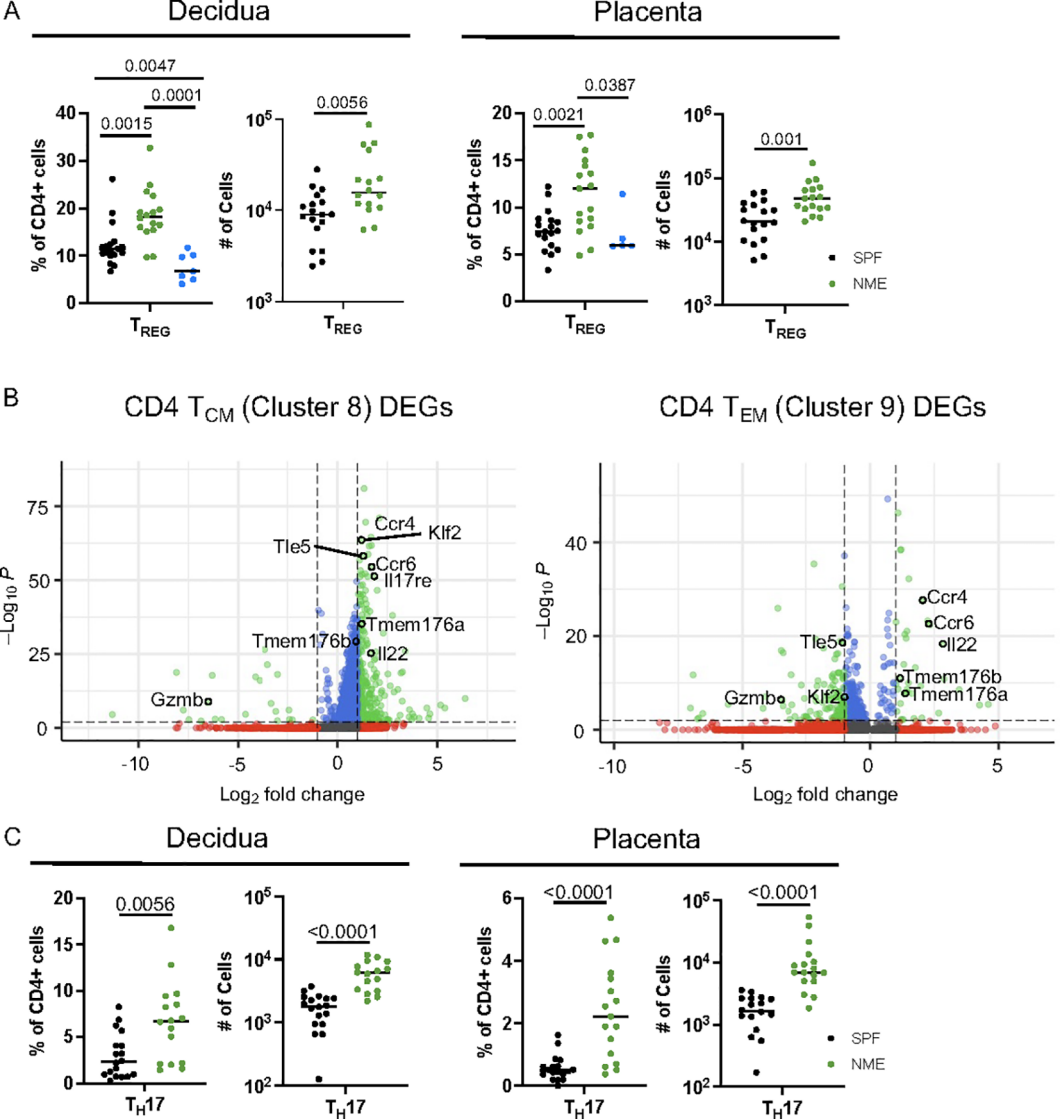
NME expands activated CD4 T cell at the MFI. **(A)** Proportion and number of T_REGs_ from SPF (black), NME (green) and human (blue) decidua (left) and placentas (right) determined flow cytometrically. (n = 16-18, three separate litters). **(B)** DEGs from scRNAseq data for CD4 T_CM_ (cluster 8) and CD4 T_EM_ (cluster 9). **(C)** Proportion and number of T_H_17 cells from SPF (black) and NME (green) in decidua (left) and placenta (right) determined flow cytometrically (n = 16-18, three separate litters).

CD4 T cell populations have been extensively studied in murine and human pregnancy with a predominant focus on T_REG_ function (6, 9, 29). In contrast to CD8 T cells, which are virtually absent in the MFI of SPF dams, CD4 T_REG_ have been found and studied in detail in SPF dams (7, 50, 51). Surprisingly, the scRNAseq data analysis suggests that the frequency of CD4 T_REG_ may be decreased in the placenta of NME dams compared to SPF dams (**Figure 3F**). In contrast, HDFC showed an increase in the proportion of T_REGs_ in both the decidua and placenta of NME dams relative to SPF dams and human samples (**Figure 6A**). Enumeration of the T_reg_ numbers in SPF and NME dams confirms the increase in T_REG_ in NME dams (**Figure 6A**). To define differences in memory CD4 T cells between SPF and NME dams, we selected memory CD4 T cell clusters 8 and 9 for DEG analysis. Both memory CD4 T cell types that were enriched by NME expressed high levels of transcripts associated with T_H_17 cells (*Rorc*, *Tmem176a*, *Tmem176b*, *Il17a*, *Il17re*, *Ccr4*, *Ccr6*, and *Il22*) (**Figure 6B**). CD4 T_CM_ (Cluster 8) was further defined by the expression of *Sell*, *Klf2* and *Tle5*, while the decreased expression of these genes in cluster 9 is consistent with an effector memory phenotype (**Figure 6B**). HDFC analysis confirmed NME placenta and decidua contained greater numbers of T_EM_ and T_CM_ CD4 memory and T_H_17 cells (**Figures 3G, H** and **6C**). Thus, natural microbial exposure expands T_REG_ numbers and activates CD4+ memory T cell populations at the MFI.

### Maternal Microbial exposure increase expression of killer lectin-like receptors on T cells

**Figure 7:**
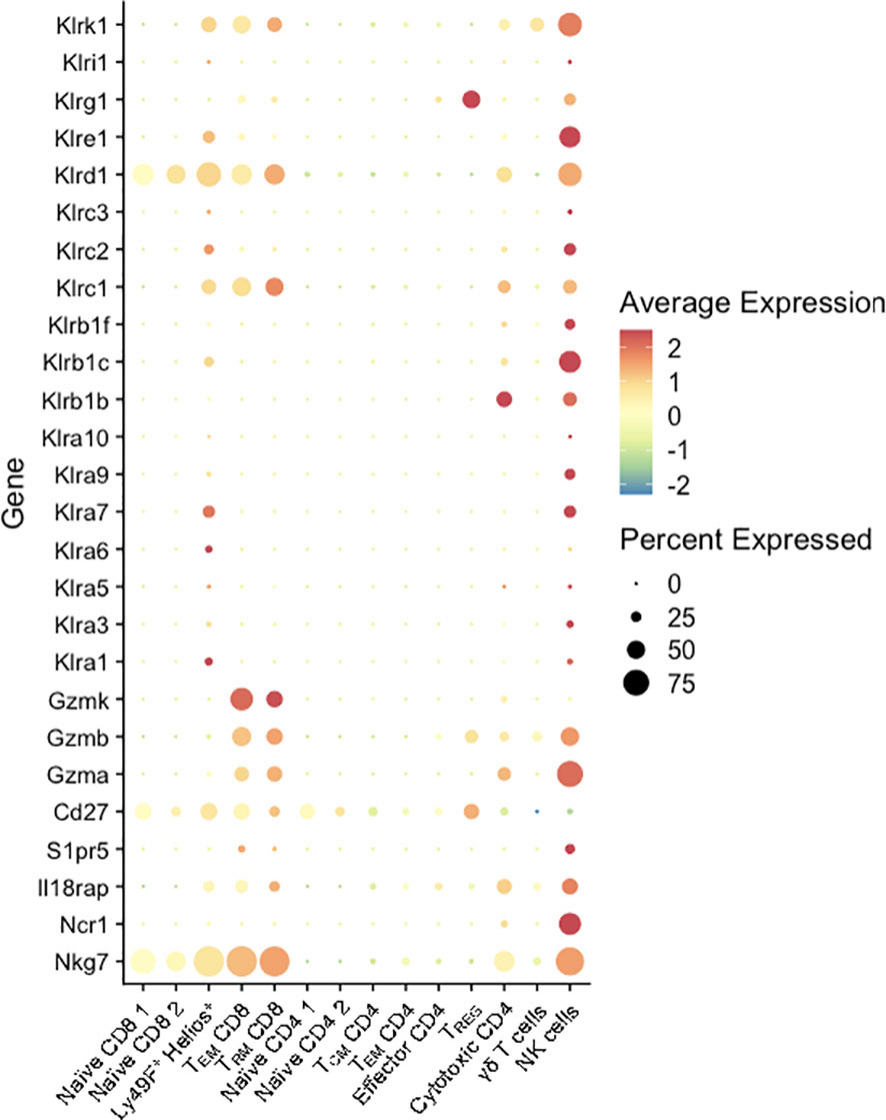
KLR and NK marker expression by T cells at the MFI. Percent expressed (size of the circle) and average expression (based on color spectrum) of select KLR genes and markers associated with NK cells across all T cell clusters from scRNAseq data determined using Seurat.

Notably, the NME-expanded CD8 T cell populations at the MFI expressed high levels of multiple genes commonly associated with natural killer cells. Previously, we observed higher levels of Natural Killer Receptors (NKRs) including Killer Immunoglobulin-like Receptors (KIRs), CD94-NKG2A (inhibitory), and CD94-NKG2C (activating) receptors on human decidual T cells than peripheral blood T cells (52, 53). In mice, Ly49 receptors or killer lectin-like receptors (KLRs), while structurally dissimilar, have evolved similarly to human NKRs and also recognize MHC class I molecules to promote or inhibit NK and CD8 T cell cytotoxicity. The scRNAseq data presented here identified multiple activating and inhibiting KLRs upregulated in MFI CD8 (clusters 3, 4, 5), and CD4 T cells (cluster 12) (**Figure 7**). Of these receptors, NKG2A (*Klrc1*) in mice recognizes the non-classical non-polymorphic MHC class I molecule Qa-1 providing them with a receptor to recognize placental trophoblasts; whereas NKG2D (*Klrk1*) binds RAE1 (*Rea-1*), H60 (*H60*), and MULT-1 (*Ulbp1*) in mice, NKG2D binds MICA/B in humans, which are upregulated on stressed or infected target cells and provide strong pro-cytolytic signaling. Thus, maternal microbial exposure activates T cells at the MFI and upregulates their potential for TCR-independent recognition of trophoblasts as well as infected or stressed target cells. Altogether, these data demonstrate that NME conditions expand T cell diversity that phenocopies human MFI T cells and provide maternal-fetal tolerance and anti-microbial responses (2, 19–22, 54, 55).

## Discussion

Using an innovative model of natural microbial exposure during murine pregnancy, we demonstrate the profound effects of microbial exposure driving increased T cell activation, differentiation, and tissue residency at the maternal-fetal interface. One of the profound changes observed was the significant increase in CD4 and CD8 memory T cells, whereas T_REG_, NK, and B cell populations had fewer differences between SPF and NME dams. Most notably, NME conditions reduced frequencies of naïve T cells, significantly expanded CD8 T cell numbers and memory CD8 and CD4 T cell diversity with signatures of activation and dysfunction unique to the MFI that phenocopy signatures observed in humans. We and others have demonstrated NME enhances peripheral T_EFF_ and T_EM_ cells, which characteristically traffic through a broad range of non-lymphoid tissues (36, 37, 41). These observations are consistent with the hypothesis that T_EM_ cells have greater ability to access decidual and placental tissues through CXCR3 expression. The relative paucity of these cell types in SPF mice likely limit T cell accumulation at the MFI. However, NME conditions may also enhance T cell recruitment and differentiation by altering the expression of integrins, cytokines, and chemokines at the MFI. Once in the MFI, T cells may acquire further unique phenotypes. Whether NME alters the MFI microenvironment to augment MFI T cell differentiation needs to be investigated further.

In this study, we used syngeneic matings to focus on the effects of natural microbial exposure on the MFI immune cell states. In most cases, NME generated immune cell states in the MFI that were much more, but not completely, aligned with human MFI immune cell diversity. The remaining gap between NME and human T cell metrics may be due to additional immune activation and regulation induced by paternal alloantigen exposure that is much reduced in syngeneic breeding. In addition, the NME and SPF dams were of similar age, healthy weight, and only primary pregnancies were used. These clinical variables likely influence T cell characteristics and should be investigated in further detail. Most notably, expanding these studies to include parity as a factor driving immune activation and memory diversity as has been observed in humans would be of high interest (56, 57).

We took a microbial agnostic approach to our experimental design, choosing to focus on the diversity of microbial exposure over the presence of individual microbes. The microbial communities of pet store mice are diverse and variable, containing bacteria, fungi, viruses, and parasites. NME dams were co-housed with a single pet store mouse throughout mating and gestation. Therefore, while the microbial exposure of an individual dam may be unique from that of others in the group, it represents a very high diversity of commensal and pathogenic microbial communities. Data from our group and others using microbial naturalization models demonstrate that the effects of diverse microbial exposure are notably consistent and generally have a similar deviation as SPF mice (36, 37, 39–41, 58). While it is entirely possible that individual microbes have specific effects on the immunology of the MFI, our data shows remarkable stability across litters co-housed with different pet store mice, and we have not identified any obvious immunological effects associated with a particular microbe.

The placenta and decidua are unique in that they are temporary tissues; as a result, markers of tissue residency are difficult to define. A large proportion of human decidual CD8 T cells are described as T_RM_ based on expression of CD69, CD103, and CD49A, however, residency markers can be tissue dependent (19, 22). Here, we demonstrate that decidual T cells are consistent with this convention, as IV– decidual T cells have a resident transcriptomic profile and express higher levels of CD69, CD103, and CD49a in the decidua. We also describe placental T cells that, while in contact with the circulation, express features that indicate they are unique from circulating T cells and may be resident in the placenta. For example, we observed IV+ placental T cells expressing higher levels of CD103 than T cells in the blood. E-cadherin, a ligand for CD103, is strongly expressed in the placental labyrinth and chorionic plate, supporting a placental tissue-specific role for CD103 in T cell homing. KLF2 is a transcription factor that regulates migration of T cells by inducing the expression of S1PR1, which permits egress from lymphoid tissues via binding to S1P. Therefore, KLF2 is considered a marker of circulating T cells, i.e., naïve and central memory T cells. Unexpectedly, high *Klf2* expression was associated with the T_EM_ population (*Sell* low *Cd44*+ cluster 4) at the MFI. However, S1P is expressed in the placenta; therefore, KLF2 expression may be important for T cell migration to and retention in the MFI (59). Altogether, both HDFC and scRNAseq data demonstrate maternal microbial exposure increases T cells with a residency phenotype at the MFI.

To evade killing by maternal CD8 T cells, human fetal trophoblasts reduce the expression of HLA-A and HLA-B in favor of HLA-C and non-classical HLA-E and HLA-G, which are thought to induce tolerance in T cells via modulation of myeloid cells or direct engagement of inhibitory NK cell receptors or KIRs (10, 52). In mice, the non-classical MHC molecules Qa-1b and Qa-2 are expressed on trophoblasts. Qa-1b is a homologue of HLA-E and provides surveillance of antigen processing defects (60). Qa-2 is proposed as a functional homologue to human HLA-G and involved in immune tolerance (61). We have previously demonstrated KIR expression is increased in human MFI CD4 and CD8 T cells relative to peripheral T cells (52). Here, we discovered high levels of KLR (Ly49) expression (similar function to human KIRs) in subsets of murine CD4 and CD8 T cells at the MFI. The *Klra6* (Ly49F)+ *Ikzf2* (Helios)+ cluster 3 exhibited similarities to intraepithelial lymphocytes (IELs) and appeared to make up a smaller proportion of T cells under NME conditions, as did the cytotoxic CD4 T cells cluster (*Klrb1*+ *Gzma*+). Conversely, memory CD8 T cells in the MFI (clusters 4 and 5) were expanded by NME conditions and expressed multiple cytotoxic molecules and a mix of activating and inhibiting KLRs. Whether the expression of KLRs increases the potential for TCR-independent recognition of infected and stressed cells or modulates T cell activity to maintain tolerance or enhance cytotoxicity requires further investigation.

In addition to activated CD8 T cells, maternal microbial exposure also enhanced activated CD4 T cell subsets at the MFI. T_REGs_ are critical for restraining active immune responses and maintaining tolerance at the MFI (6, 11, 62). Unsurprisingly, HDFC analysis showed T_REGs_ increased in number and proportion at the MFI in response to maternal immune activation, presumably to regulate active immune cells and maintain tolerance. This was not evident in the scRNAseq data, likely owing to differences in sensitivity to detect bona fide T_REGs_ between the two methods. T_H_17 cells were found to be preferentially expanded by NME conditions at the MFI in both scRNAseq and HDFC datasets. T_H_17 cells secrete proinflammatory cytokines (IL-17, IL-21, IL-22, and IL-26) to assist in the defense against extracellular bacteria and fungi (63). Overactive T_H_17 responses can lead to inflammation and tissue destruction and are linked to several pregnancy complications (e.g., preeclampsia, gestational diabetes mellitus, and preterm birth) (64). T_H_17 cells can also play a pathologic role in allograft rejection as the balance between T_H_17 and T_REG_ cell responses is crucial in reducing the risk of rejection (65, 66). While the expansion of T_REGs_ at the MFI may be sufficient to control cytotoxic and T_H_17 cells, we also observed a corresponding expansion of *Il23r* expressing γδ T cells, which have been described to identify a subset of γδ T cells that suppress T_H_1 and T_H_17 cells by absorbing the available IL-23 (67). Altogether, maternal microbial exposure enhanced anti-microbial and immune regulatory T cell populations providing insight into the cellular mechanisms that balance host defense and tolerance at the MFI in the context of physiological microbial exposure and a healthy pregnancy.

Our data demonstrate maternal microbial exposure dramatically enhanced T cell heterogeneity to replicate the T cell landscape of the human MFI more accurately. However, there still exist differences between human and NME mouse MFI T cells. While we observed that NME increases the number and proportion of T cells at the MFI, T cells only comprised ~6% of immune cells at 14.5 dg, well below the 20-60% at the human MFI at the beginning of the third trimester (3, 17, 68). This could reflect differences in the length of gestation between humans and mice, as memory T cells accumulate in the MFI over time. Differences in the anatomy of human and murine MFI and the average number of concepti per pregnancy are limitations of the model that are not feasibly altered but may also impact MFI T cell immunology.

Cumulatively, this study presents an improved model of human pregnancy, creating opportunities for detailed mechanistic studies of microenvironmental signals driving T cell function and recruitment. The extent to which maternal microbial exposure alters T cell influx and diversity to the MFI is vast, and careful and comprehensive investigation into how these cells contribute to tolerance, immunity, and inflammation in healthy and pathogenic pregnancies is warranted.

## Data Availability

The datasets presented in this study can be found in online repositories. The names of the repository/repositories and accession number(s) can be found below: https://www.ncbi.nlm.nih.gov/, GSE293451.

## Supplementary material

Supplementary Figure 1 Example H&E staining of NME and SPF MFI frozen sections. Images are labeled with maternal (m) and fetal (f) sides.

Supplementary Figure 2 Frequency of IV labeled immune cells in the placenta and decidua. **(A)** Example CD45.2 IV+ labeling of leukocytes from decidua, placenta, and maternal blood. **(B)** Proportion of IV+ leukocytes in decidua, placenta, and maternal blood (n = 10–12 MFI from 4 separate litters). **(C)** Example CD45.2 IV+ labeling of T cells from decidua, placenta, and maternal blood. **(D)** Proportion of IV+ T cells in decidua, placenta, and maternal blood (n = 10–12 MFI from 4 separate litters).

Supplementary Figure 3 Leukocyte composition at the MFI. **(A)** Top ten DEGs for each scRNAseq leukocyte cluster. **(B, C)** Cytometric enumeration of NK cells, CD8 T cells, CD4 T cells, B cells, and macrophages in the decidua **(B)** and placenta **(C)**.

Supplementary Figure 4 NME conditions associated with increased Jun expression. Volcano plot of DEGs from **(A)** Macrophage clusters, **(B)** B cell clusters, **(C)** NK cell clusters.

Supplementary Figure 5 Example flow cytometry gating strategy. **(A)** gating strategy used to identify T cells populations. **(B)** gating strategy used for FACS sorting of CD45+ IV+ and IV- leukocytes in preparation for single cell sequencing.

Supplementary Figure 6 T cell composition at the MFI. **(A)** Top ten DEGs for each scRNAseq T cell cluster. **(B, C)** Cytometric enumeration of T cell subsets in the decidua **(B)** and placenta **(C)**.
