# Supplementary figures and images for: Natural microbial exposure populates the maternal fetal interface with diverse T cells
Source: Front Immunol. 2025 Jul 9;16:1616491. doi: 10.3389/fimmu.2025.1616491 (PMC12283277; doi:10.3389/fimmu.2025.1616491)

NME

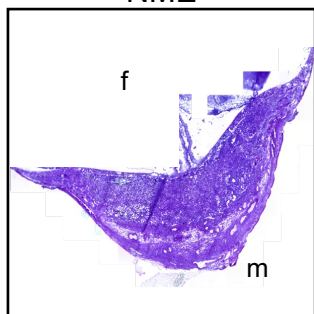

SPF

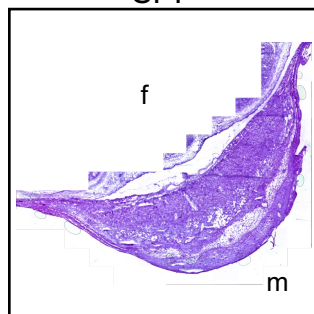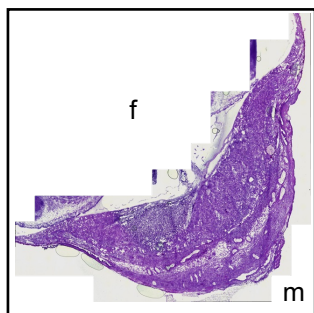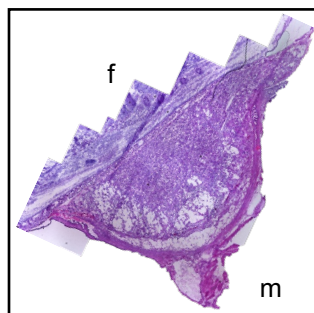

Supplement: Supplementary Figure 1 — Example H&E staining of NME and SPF MFI frozen sections. Images are labeled with maternal (m) and fetal (f) sides. [file DataSheet1.pdf]

# Leukocytes

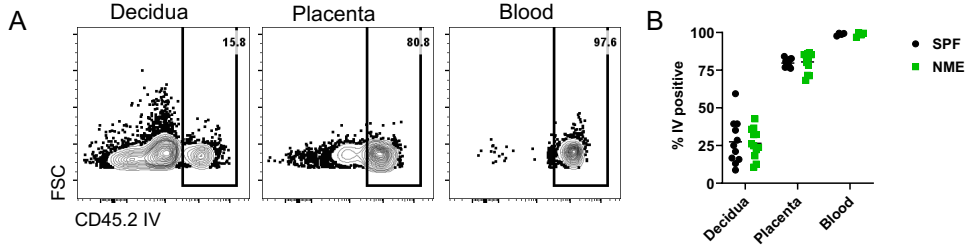

# T cells

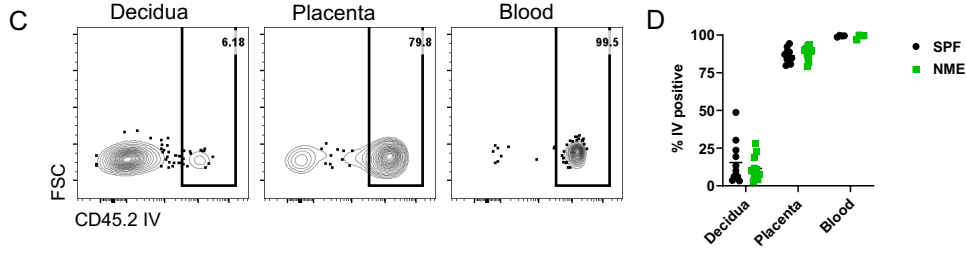

Supplement: Supplementary Figure 2 — Frequency of IV labeled immune cells in the placenta and decidua. (A) Example CD45.2 IV+ labeling of leukocytes from decidua, placenta, and maternal blood. (B) Proportion of IV+ leukocytes in decidua, placenta, and maternal blood (n = 10–12 MFI from 4 separate litters). (C) Example CD45.2 IV+ labeling of T cells from decidua, placenta, and maternal blood. (D) Proportion of IV+ T cells in decidua, placenta, and maternal blood (n = 10–12 MFI from 4 separate litters). [file DataSheet2.pdf]

A

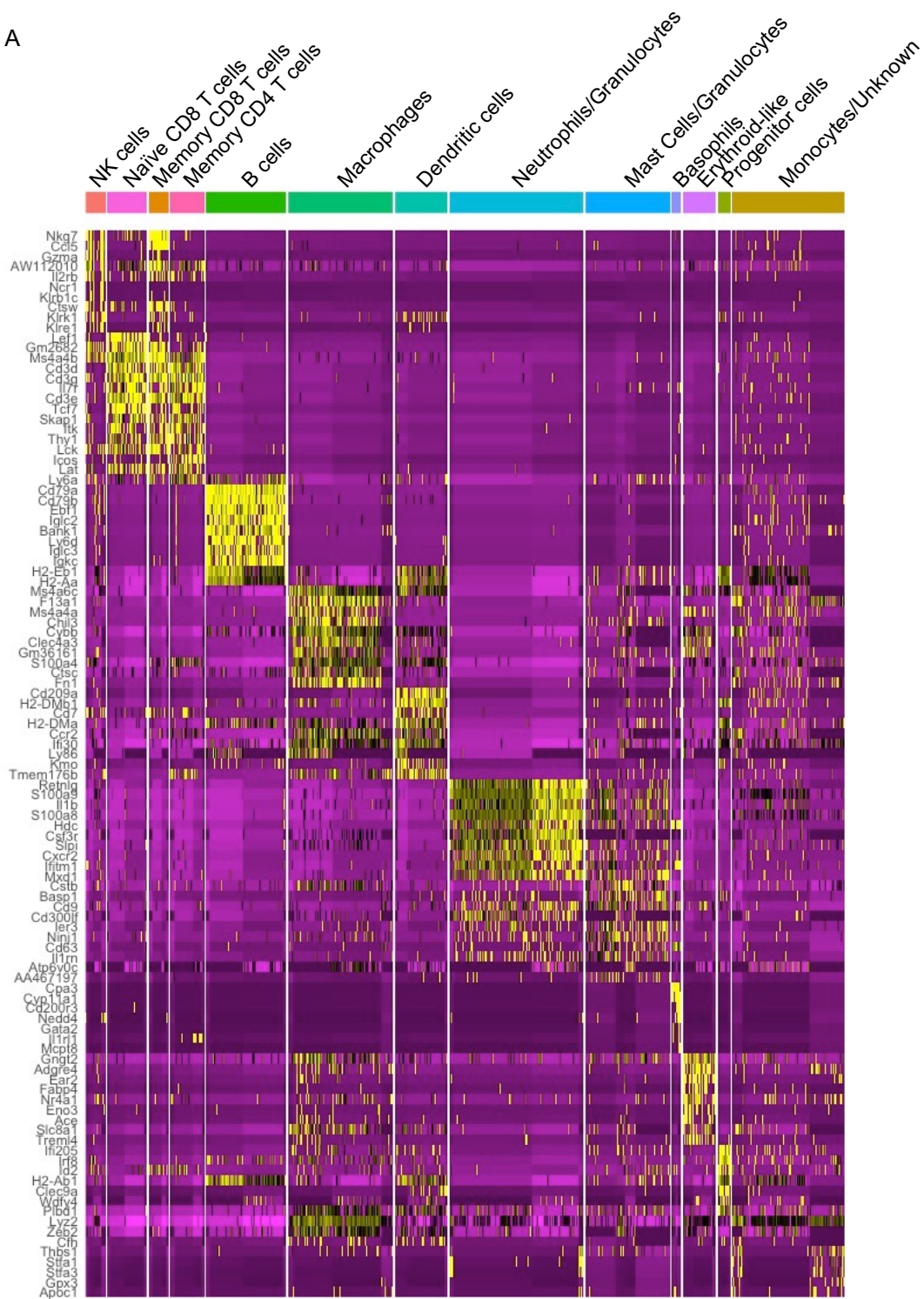

B

HDFC: Decidua

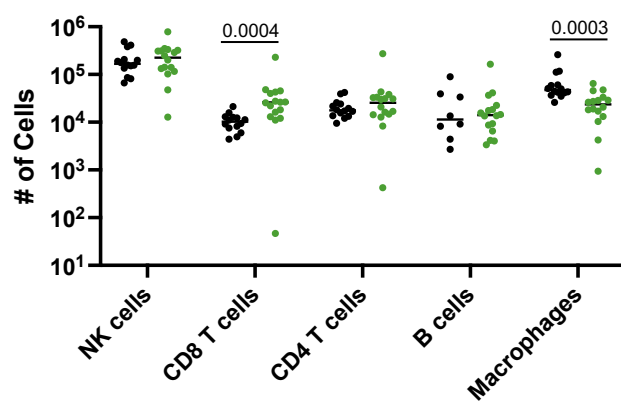

C

HDFC: Placenta

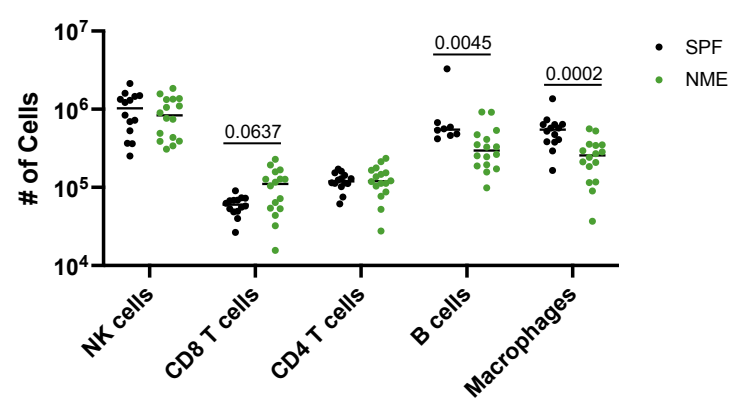

Supplement: Supplementary Figure 3 — Leukocyte composition at the MFI. (A) Top ten DEGs for each scRNAseq leukocyte cluster. (B, C) Cytometric enumeration of NK cells, CD8 T cells, CD4 T cells, B cells, and macrophages in the decidua (B) and placenta (C). [file DataSheet3.pdf]

**A** NME vs. SPF Macrophage clusters

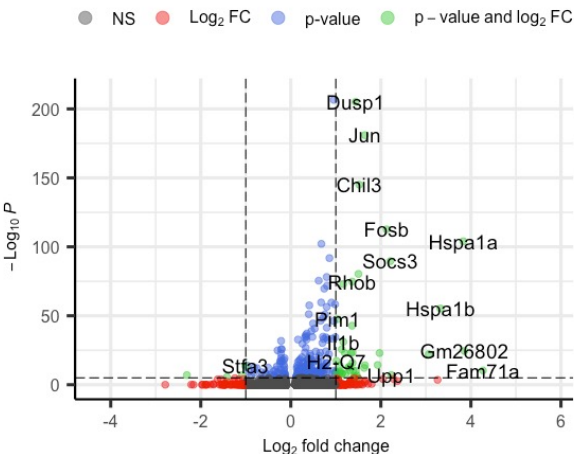

**B** NME vs. SPF B cell clusters

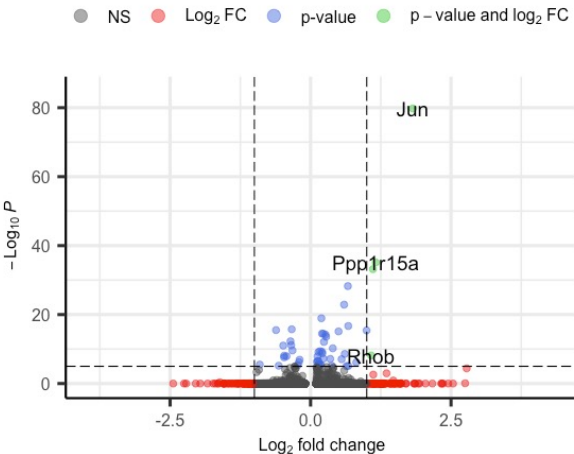

**C** NME vs. SPF NK cell cluster

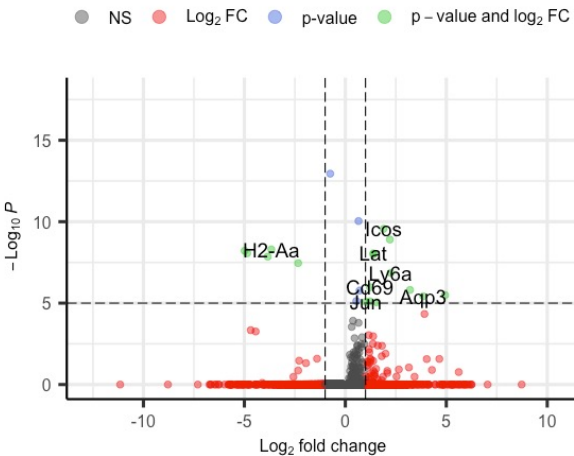

Supplement: Supplementary Figure 4 — NME conditions associated with increased Jun expression. Volcano plot of DEGs from (A) Macrophage clusters, (B) B cell clusters, (C) NK cell clusters. [file DataSheet4.pdf]

A

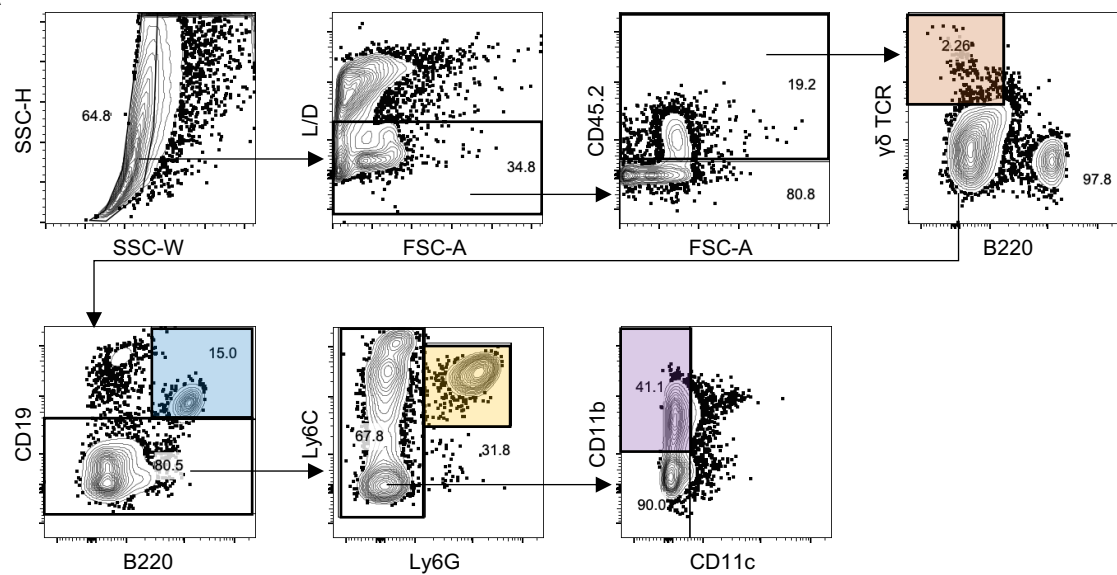

B

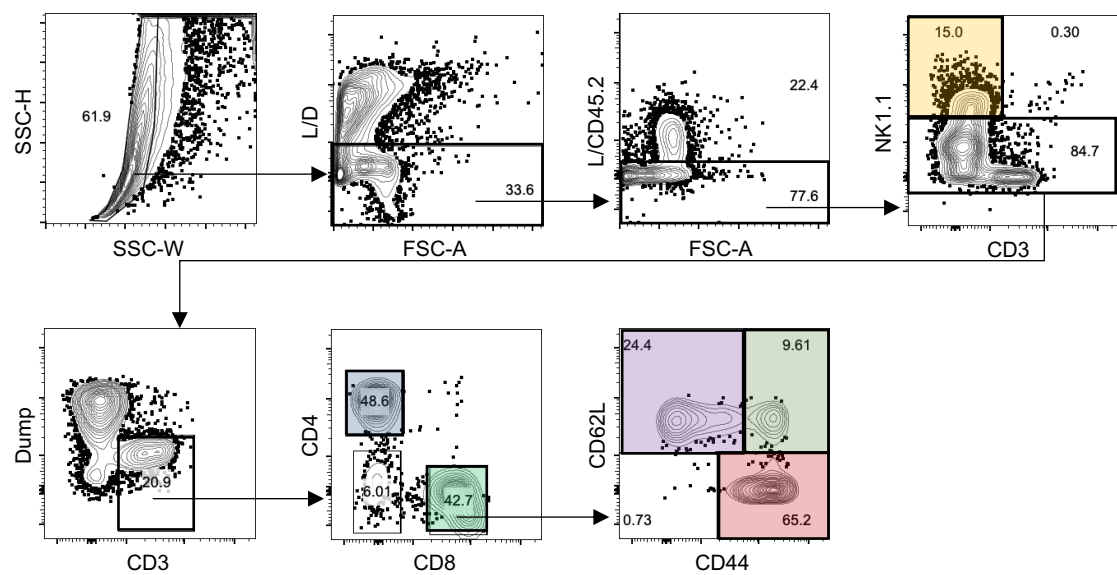

C

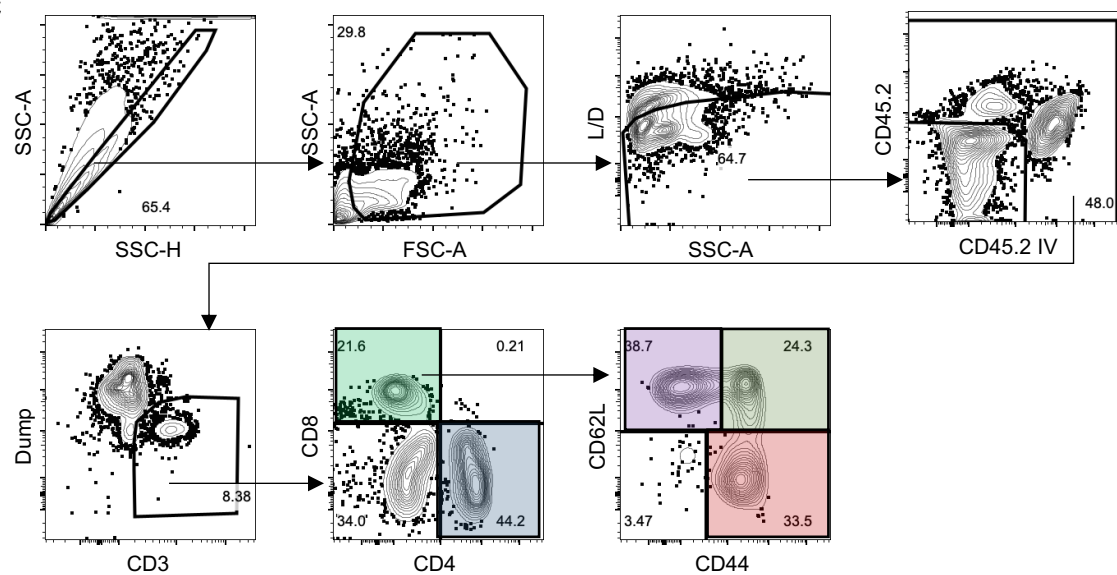

Supplement: Supplementary Figure 5 — Example flow cytometry gating strategy. (A) gating strategy used to identify T cells populations. (B) gating strategy used for FACS sorting of CD45+ IV+ and IV- leukocytes in preparation for single cell sequencing. [file DataSheet5.pdf]

A

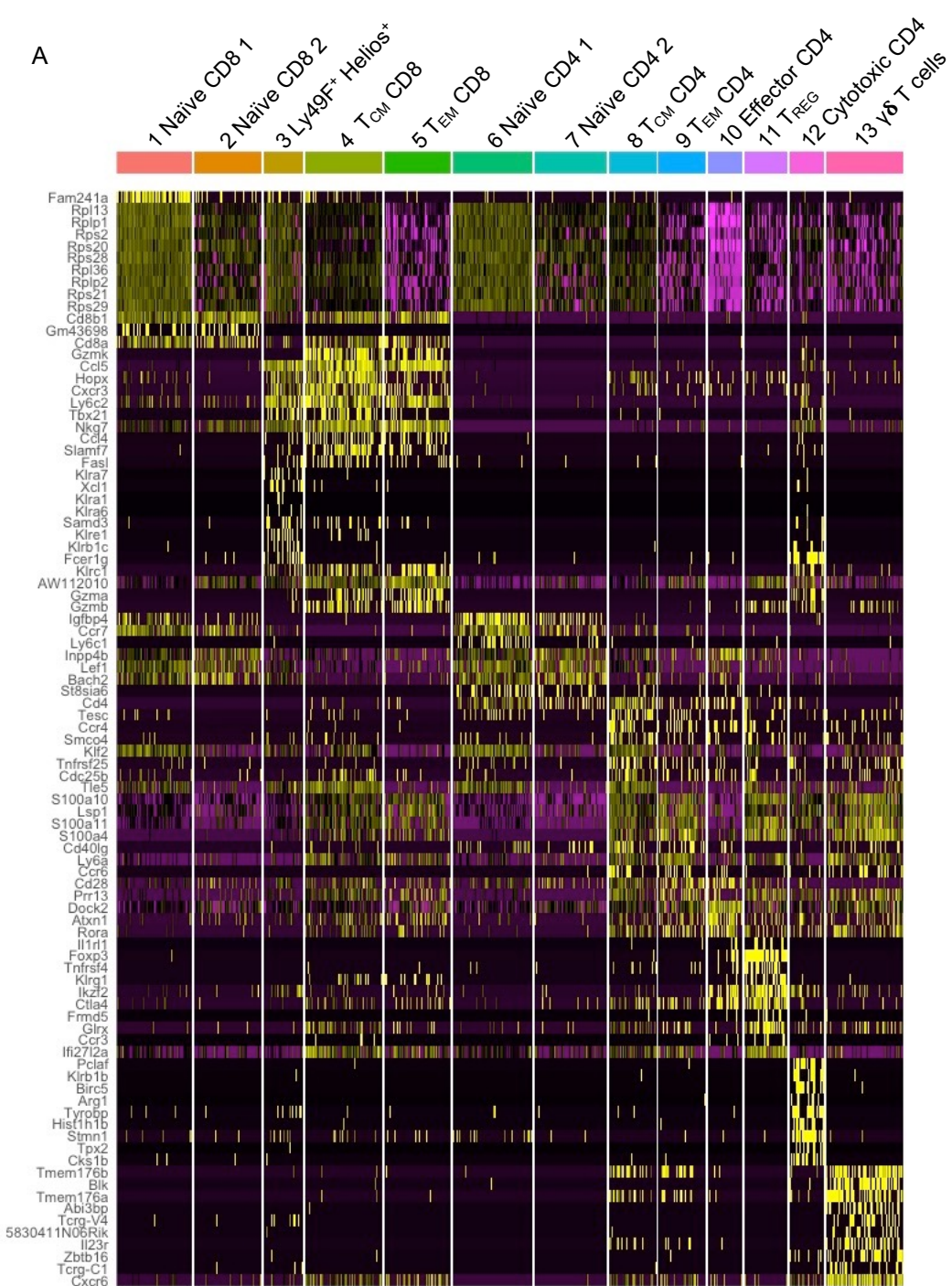

B

HDFC: Decidua

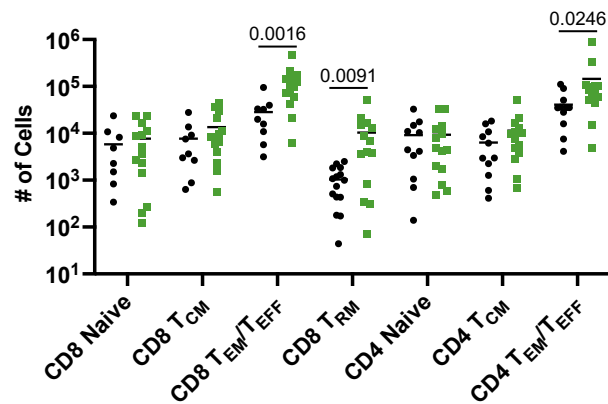

C

HDFC: Placenta

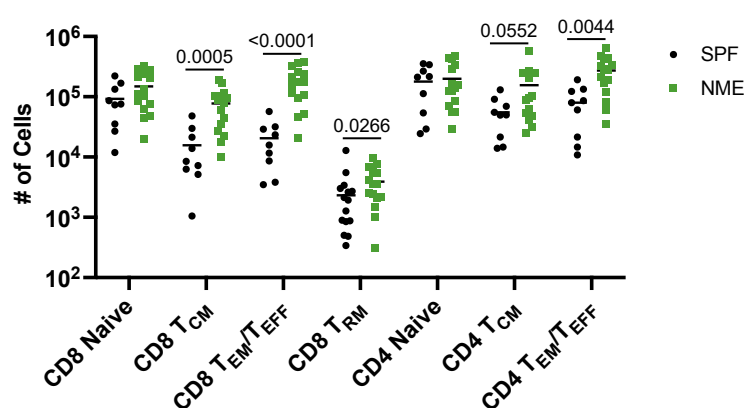

Supplement: Supplementary Figure 6 — T cell composition at the MFI. (A) Top ten DEGs for each scRNAseq T cell cluster. (B, C) Cytometric enumeration of T cell subsets in the decidua (B) and placenta (C). [file DataSheet6.pdf]
